# Correlating confocal microscopy and atomic force indentation reveals metastatic cancer cells stiffen during invasion into collagen I matrices

**DOI:** 10.1038/srep19686

**Published:** 2016-01-27

**Authors:** Jack R. Staunton, Bryant L. Doss, Stuart Lindsay, Robert Ros

**Affiliations:** 1 Department of Physics, Arizona State University, Tempe, AZ 85287; 2 Center for Biological Physics, Arizona State University, Tempe, AZ 85287; 3 Biodesign Institute, Arizona State University, Tempe, AZ 85287; 4 Department of Chemistry and Biochemistry, Arizona State University, Tempe, AZ 85287.

## Abstract

Mechanical interactions between cells and their microenvironment dictate cell phenotype and behavior, calling for cell mechanics measurements in three-dimensional (3D) extracellular matrices (ECM). Here we describe a novel technique for quantitative mechanical characterization of soft, heterogeneous samples in 3D. The technique is based on the integration of atomic force microscopy (AFM) based deep indentation, confocal fluorescence microscopy, finite element (FE) simulations and analytical modeling. With this method, the force response of a cell embedded in 3D ECM can be decoupled from that of its surroundings, enabling quantitative determination of the elastic properties of both the cell and the matrix. We applied the technique to the quantification of the elastic properties of metastatic breast adenocarcinoma cells invading into collagen hydrogels. We found that actively invading and fully embedded cells are significantly stiffer than cells remaining on top of the collagen, a clear example of phenotypical change in response to the 3D environment. Treatment with Rho-associated protein kinase (ROCK) inhibitor significantly reduces this stiffening, indicating that actomyosin contractility plays a major role in the initial steps of metastatic invasion.

Cell behavior is guided by the three-dimensional (3D) microenvironment^1^,^2^,^3^,^4^. Reciprocal mechanical interactions between cells and their microenvironment can dictate cell phenotype and behavior, requiring studies of cells in physiologically relevant 3D extracellular matrices (ECM)^3^,^5^,^6^,^7^,^8^,^9^. In cancer development, cell stiffness has been demonstrated to be a key indicator of metastatic potential. Several studies indicate that cancer cells of various types are more compliant than their ‘normal’ counterparts^10^,^11^,^12^,^13^,^14^,^15^,^16^,^17^. Deformability was shown to correlate positively with malignancy among pre-cancerous esophageal cells^18^. MDA-MB-231 metastatic breast cancer cells were found to bemore deformable than non-tumorigenic MCF-10A mammary epithelial cells^11^,^15^,^16^,^17^, depending on both indentation depth and the subcellular region of the cell indented^15^,^16^. In principle, these aberrant mechanical features could be exploited in diagnosis or prognosis (e.g. in conjunction with high-throughput ‘fluid biopsy’ screening of circulating tumor cells) and provide novel cytoskeletal targets in anti-metastatic drug design. Promising approaches for diagnostics are novel high-throughput techniques for mechanical profiling^19^,^20^. Recently, Plodinec *et al.* demonstrated that AFM indentation can be used for nanomechanical profiling of biopsy samples for clinical diagnostics of breast cancer^21^.

Cell motility in 3D microenvironments depends upon the mechanical interplay between the cell and ECM. Cell elasticity is closely related to cell motility^22^, but elasticity measurements during cell migration are lacking. Cancer cells invading 3D matrices can exhibit multiple modes of single-cell motility, variously featuring polarized or non-polar morphology, pseudopodia, lamellipodia, filopodia, lobopodia, invadopodia or membrane blebs, secretion of proteolytic factors, and formation of cell-ECM contacts via integrins or other adhesion receptors^23^,^24^. Many of these processes are regulated by Rho, Rac and Cdc-42 GTPases^25^. For migration through narrow channels, an alternative migration mechanism based on differential water permeability at the leading and tailing edge of the cell has been proposed^26^. Cell motility is also interrelated with mechanical properties of the surrounding matrix. Cancer cells may switch or blend between these modes depending on environmental factors such as ligand type and density, cross-linking, matrix porosity, and stiffness^27^. 3D particle tracking of migrating tumor cells in 3D collagen gels has shown elastic deformation of the matrix at the leading edge and irreversible matrix ruptures at the trailing edge^28^. A similar approach allowed the determination of strain patterns in the matrix around single invading MDA-MB-231 cells^29^,^30^. Measuring cell and ECM mechanical properties concomitantly during cell migration can therefore provide needed insights into the mechanisms of metastatic invasion.

However, most quantitative single cell deformation measurements to date have been conducted on cells either in suspension or adherent to tissue culture substrates^10^,^11^,^12^,^13^,^14^,^15^,^16^,^17^. Cell mechanics measurements in 3D environments are very rare. Wirtz *et al.* developed a microrheology technique based on intracellular particle tracking that probes the cytoplasmic viscoelasticity^31^. This technique has been applied to breast cells with increasing metastatic potential in collagen gels, indicating a correlation between cytoplasmic stiffening and metastatic potential^32^. Recently, Kamm *et al.* used mitochondria-tracking microrheology and Brownian dynamics simulations to compare intracellular mechanics in 2D and 3D^33^. They found for MDA-MB-231 cells in 3D environments more solid like internal motions compared to cells in 2D. Further, Guo *et al.* combined intracellular particle tracking with active optical tweezers based microrheology to quantify random forces in the cytoplasm^34^. This novel technique allows study of stochastic motor protein activity in living cells. In all of these experiments, the mechanical properties and forces of the cell are determined from within the cytoplasm. The mechanical properties of the cell as a whole reflect contributions from the nucleus, microtubule and intermediate filament networks, actin cytoskeleton, membrane, and are additionally influenced by interactions with the pericellular ECM. Mechanics of the actin cortex and membrane, which can be probed in 2D environments by increasingly well-established and available AFM indentation methods, are especially important for deeper understanding of 3D cell-matrix interactions. A method that expands the applicability of AFM indentation to quantify the deformability and mechanical properties cells in 3D microenvironments from the outside would capture these cortical and cytoskeletal contributions, and complement other techniques that probe the cell from within the cytoplasm.

Quantifying deep indentations into heterogeneous samples remains a challenge. When an indentation induces a deformation field in a mechanically heterogeneous sample, the force response will reflect this^35^. A new framework for approximating these effects is needed in order to decouple the mechanical properties of distinct components, such as embedded cells and the pericellular ECM surrounding them. Here we describe a novel technique allowing such a deconvolution of mechanical properties of heterogeneous materials. We then quantified the Young’s moduli of metastatic breast adenocarcinoma MDA-MB-231 cells invading into bovine collagen I hydrogels. We studied the influence of i) different invasion depths (fully and partially embedded cells); ii) variations of matrix stiffness; and iii) the influence of ROCK inhibition on cell stiffness during invasion.

## Results

### AFM indentation with mesoscopic sphero-conical probes

**Figure 1:**
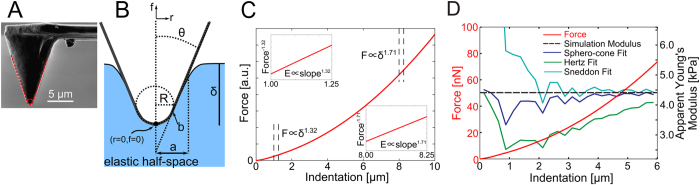
AFM indentation with a sphero-conical probe. (**A**) Scanning electron microscope image of a sphero-conical AFM probe. The red annotation shows the outline of the probe geometry with a radius of R = 695 nm and a cone with a semi-vertical angle θ = 18.8°). (**B**) Diagram of a sphero-conical probe indenting an elastic half-space. (**C**) Linearization fitting method of a theoretical force-indentation curve from a sphero-conical probe. The power-law dependence is calculated to produce linearized force-indentation curves as shown in the insets (y-axis not to scale). (**D**) Force-indentation curve of a sphero-conical tip (R = 750 nm, θ = 22.5°) indenting into a homogeneous elastic half-space (E = 4.41 kPa, ν = 0.47, displayed in black, right axis) generated with finite element analysis (red, left axis) and the corresponding piecewise depth-dependent fits using the sphero-conical model (blue, right axis), Hertz model (green), and Sneddon model (purple).

Indentation of cells embedded in 3D ECM requires probes with a sufficient height to deform the surface several micrometers without coming into contact with the cantilever. Large spherical probes can indent sufficiently deep, but their contact profile would reduce spatial resolution and force sensitivity due to the stiffer cantilevers required. Therefore, to quantify mechanical properties of cells in collagen matrices, we applied AFM force-indentation measurements with mesoscopic sphero-conical probes (tip radii ~ 700 nm) (Fig. 1A). These probes allow deep, unobstructed indentation of the tip with a relatively high lateral resolution in comparison to large spherical probes. The tips feature a well-defined contact geometry compared to sharp commercial probes, described by a sphere of radius *R* transitioning into a cone with a semi-vertical angle *θ* (Fig. 1B). The most commonly used contact models for AFM indentation experiments, the parabolic and conical models, account for only one of these parameters. The hyperbolic^36^ and Briscoe blunted cone^37^ models incorporate both *R* and *θ*; however the hyperbolic model exhibits an extremely gradual transition which results in an increased radius of contact, while the Briscoe model contains a discontinuity in the curvature of the tip which is not ideal for the length scale of our measurements (μm). So, on the basis of the Sneddon’s method^38^, we derived a non-adhesive elastic contact model for such a “sphero-conical” indenter geometry (Fig. 1B). The end result and derivation is shown in detail in the Materials and Methods section.

Briefly, Sneddon demonstrated that the equilibrium solution for the distribution of stress along the boundary of a linear elastic half-space deformed normally by a rigid axisymmetric punch is a mixed boundary value problem that (by employing Hankel transform theory) can be reduced to the solution of dual integral equations giving the force *F* and distance *δ* of indentation, respectively, in terms of the sample Young’s modulus 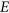, Poisson ratio *ν*, and the punch profile^38^. By appropriate transformation of variables these can be solved for a punch of arbitrary (but still axisymmetric) profile described by a function *f* in cylindrical coordinates. The smoothly transitioning sphero-conical profile requires a two-piece piecewise function that is smooth and continuous at the transition point *b* (Fig. 1B). The integral equations must therefore be solved in two regimes for *a* ≤ *b* and *b* < *a*, where *a* is the contact radius and *b* is the location at which the punch transitions from the spherical to conical regime. For indentation depth *δ* << *R* (where *R* is the tip radius), the resulting relation is similar to standard models for spherical indenters, e.g. the Hertz model^39^. When *R* = 0, the equations reduce to those of the Sneddon model for a conical indenter^38^. For a given *R* and *θ*, the result is very similar to that given by Briscoe for a blunted cone^37^.

To determine the Young’s modulus from experimental AFM force-indentation curves, we adopt a fitting procedure (described by Eqs. 1, 2, 3, 4, 5 in the Materials and Methods section) in which the force-indentation relation is transformed (Fig. 1C). The resulting curve is segmented into bins of indentations depth and each segment is fit by piecewise linear regression to yield the depth-dependent *apparent Young’s modulus*. For example, conical indenters follow a power law 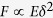 (for spherical indenters, 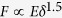). Rearranging the relationship, 
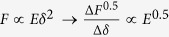
 gives the curve 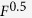
vs. 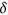 which is then segmented into (e.g. 250 nm) bins, and the slope of each segment is found to give the apparent Young’s modulus at the corresponding depth. Similar procedures have been used in analysis of Hertz model indentations and have the benefit of reducing errors in extracting the Young’s modulus due to uncertainty in determining the contact point^18^,^40^,^41^. For sphero-conical indenters, the power law exponent varies with depth, ranging from 1 (for small *θ*) to 2 (for deep indentations or large *θ*), with shallow indentations being nearly 1.5.

Because this regression method is local in nature, it is more sensitive to sample heterogeneity and may therefore be used to study the depth-dependence of the force response by segmenting the force curve into smaller indentation bins. If the sample is perfectly elastic and homogeneous, we observe little or no depth dependence. If not, then the deviations are apparent, rather than being hidden by a poor global fit. Note, however, that the apparent Young’s modulus *does not* account for the effects of underlying or superficial material on the mechanical response of material at a given depth, although these effects may be observed in the apparent Young’s modulus. For that, other approaches, described below, are required.

To test the accuracy of the newly derived sphero-conical contact model, we used finite element (FE) analysis. We performed FE simulations of an indentation using a sphero-conical tip onto an ideal elastic half-space with imposed Young’s modulus and Poisson ratio. Each simulated indentation generates a virtual indentation curve of *F*-*δ* coordinates. These curves were then fit piecewise to yield the apparent Young’s modulus as described above, using the equations for contact models corresponding to a variety of different tip shapes (Fig. 1D, Table S1). Because the sample is ideally elastic and flat, fitting the segments of the force-indentation curve in each bin should return the imposed Young’s modulus for the simulation. We observe that with both the Hertz (spherical) and Sneddon (conical) model, the depth-dependent modulus does not yield the imposed simulation modulus and instead varies with depth. However, with the sphero-conical model, it is more constant and accurate with depth, with much smaller fluctuations that are attributable to finite element artifacts from meshing or other numeric factors. These simulations illustrate the improvements in accuracy for applying an analytical model with the correct contact geometry.

### Correlated AFM nanoindentation with confocal laser scanning microscope (CLSM) on cells in 3D collagen I matrixes

**Figure 2:**
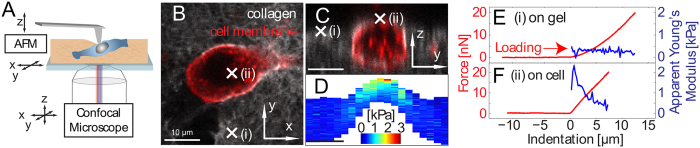
Combined AFM indentation and confocal fluorescence microscopy for elasticity measurements on embedded cells. (**A**) Schematic of AFM-CLSM setup. The AFM is sample scanning in the X and Y directions, and the objective of the confocal microscope is scanned in the X, Y, and Z directions. The AFM tip is aligned in the confocal volume prior to indentations and this alignment is preserved during the experiment when the sample is scanned. (**B**) Lateral (XY) and (**C**) axial (YZ) confocal fluorescence micrographs of an MDA-MB-231 cell partially embedded in collagen. The cell membrane (red) and collagen (white) are fluorescently labeled. (**D**) Spatially co-registered elasticity map showing the apparent Young’s moduli (fitted piecewise in 250 nm intervals of indentation depth) determined from indentations in the axial plane in (**C**). (**E,F**) Force-indentation curves (red) and corresponding apparent Young’s moduli (blue) from points (i) and (ii). Scale bars in (**B–D**): 10 μm.

To determine the Young’s moduli of single cells embedded in hydrogels, we used a combined AFM and confocal fluorescence microscope setup (Fig. 2A). The use of AFM in combination with confocal microscopy has gained a lot of interest recently^42^,^43^,^44^,^45^,^46^,^47^,^48^, and commercial systems are available. Our setup allows a precise alignment of the AFM probe with the microscope objective in combination with synchronization of the two microscopes for precise determination of the location of the AFM indentation in the confocal images^18^,^48^,^49^. 2D fluorescence confocal scans are conducted in lateral (Fig. 2B) and axial (Fig. 2C) planes. 20 AFM indentations with sphero-conical tips were then conducted in the axial plane at 2 μm intervals. Every indentation curve was analyzed piecewise in 250 nm intervals, and the depth-dependent apparent Young’s moduli were calculated for each segment according to the sphero-conical contact model. These measurements result in an elasticity map (Fig. 2D) displaying the apparent Young’s modulus that is co-located with the confocal images. Figure 2E,F show two representative force-indentation curves on collagen matrix (Fig. 2E) and cellular (Fig. 2F) regions (i) and (ii), respectively, indicated by x’s (Fig. 2B,C). While the apparent Young’s modulus of the matrix is relatively constant for different indentation depths, the measurement over the cell shows a much stiffer value at shallow indentation and decreases with indentation depth, since the force response is influenced by the underlying substrate.

### Quantitative determination of the elastic modulus of heterogeneous materials

Next, we considered how well such a sample might be represented as a composite of two homogeneous elastic layers. The apparent elasticity map in Fig. 2D and the two force-indentation measurements (Fig. 2E,F) show a clear mechanical contrast between the cell and the matrix, with the matrix having a lower apparent Young’s modulus. The force response of the cantilever throughout the indentation reflects the mechanically heterogeneous components at different depths^35^. We therefore developed a framework for approximating the effect of heterogeneous materials to assist with decoupling the mechanical properties of the cell from the effects of the matrix.

**Figure 3:**
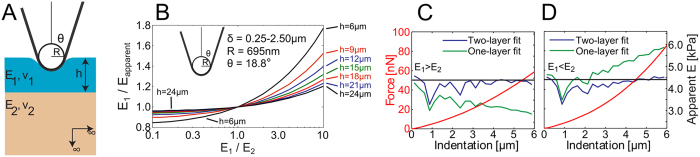
Decoupling of mechanical properties of two-layer samples. (**A**) Illustration depicting a sphero-conical tip indenting into a bonded two-layer elastic half-space. (**B**) Young’s modulus correction values calculated for the cell experiments using a sphero-conical tip with radius 695 nm, θ = 18.8°, δ = 0.25–2.50 μm for various layer heights and layer elasticity mismatches. (**C**) Force-indentation curve from finite element analysis (red) and piecewise depth-dependent fits for the Young’s modulus using the sphero-conical model with (blue) and without (green) substrate correction, and the imposed modulus of the top layer (black). For this simulation, h = 10 μm, E_1_ = 4.41 kPa, ν_1_ = 0.47, E_2_ = 1.49 kPa, ν_2_ = 0.49. (**D**) Similar to (**C**) with h = 10 μm, E_1_ = 4.41 kPa, ν_1_ = 0.47, E_2_ = 14.4 kPa, ν_2_ = 0.44.

We first examined force-indentation responses on heterogeneous materials, such as when there is a substrate underneath the sample of interest with a different elastic modulus. Dhaliwal and Rau presented a generalized analytical solution to the indentation problem of two bonded layers with different elastic properties (Fig. 3A) in the form of a Fredholm Integral Equation of the Second Kind^40^,^50^ (see Eqs. 17, 18, 19, 20, 21, 22, 23, 24, 25 in the Materials and Methods section). It is applicable to any arbitrary axisymmetric punch, including sphero-conical indenters. The equations are solved numerically to produce a substrate corrected force-indentation relationship for a given sample configuration in terms of the elastic moduli and Poisson’s ratios of the two layers (*E*_*1*_, *E*_*2*_, *ν*_*1*_, *ν*_*2*_) the thickness of the top layer *h* (the bottom layer is presumed to be infinitely thick), the tip shape, and the indentation force 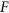 and depth *δ*.

If the indenter shape and indentation depth are known *a priori* and the Poisson ratios are either known or assumed, then there are three parameters which will affect the elastic response from indentation: *E*_*1*_, *E*_*2*_, and *h*. When two of these three parameters are known, we may use the analytic model to deduce the value of the unknown parameter by comparing the apparent Young’s modulus between the numeric results of Eqs. 17, 18, 19, 20, 21, 22, 23, 24, 25 and experimental data. The value by which the apparent Young’s modulus must be scaled to yield the substrate corrected apparent Young’s modulus is pre-tabulated and given as a function of the elasticity mismatch between the two layers (Fig. 3B). As seen in Figure 3B, substrate effects can be considerable even for shallow indentations (0.25–2.50 μm) on thick top layers, especially in the case of a stiff top layer. We then performed more FE analyses to assess the effects of an elastic mismatch between a finitely thick top layer and an underlying substrate layer (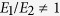) on the determination of the Young’s modulus of the top layer. Virtual indentation curves from simulated indentations of an elastic material with softer (Fig. 3C) and stiffer (Fig. 3D) substrates were fitted using the sphero-conical contact model with and without the substrate corrections given by Eq. 17, 18, 19, 20, 21, 22, 23, 24, 25. In both types of elastic mismatch, the apparent Young’s modulus changes with depth as the probe begins to feel the effects of the underlying layer. When the data is corrected for the substrate, the apparent Young’s modulus matches the imposed Young’s modulus in the finite element simulation (Fig. 3C,D, blue).

For the remaining text, we use the term ‘Young’s modulus’ to refer to the top layer corrected modulus *E*_*1*_.

### MDA-MB-231 cells stiffen as they invade collagen I matrices

**Figure 4:**
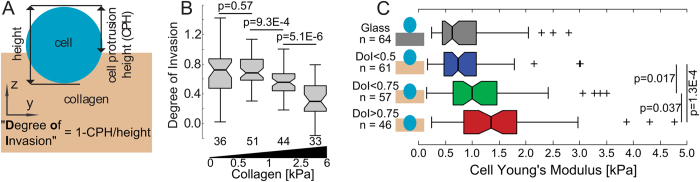
Young’s moduli of MDA-MB-231 cells on glass and partially embedded in collagen I hydrogels. (**A**) Schematic defining the degree of invasion (DoI) quantifying the amount of cell embedding. (**B**) Box plot showing the DoI for different collagen I stiffnesses, number of replicates are shown underneath. (**C**) Box plot showing the corrected cell Young’s modulus at various stages of partial invasion. *p*-values are calculated using the Mann-Whitney U test.

To investigate the interplay between cancer cell elasticity and matrix invasion, MDA-MB-231 breast cancer cells were seeded on top of fully polymerized bovine collagen I hydrogels and allowed to invade the gel for periods ranging 6−96 h before imaging and indentation, at which time many cells were either partially or fully embedded within the matrix. Within 24 h, the cells began to invade into the collagen matrices. Gels had areal porosity ~70% and mean pore area ~0.3 μm^2^ (Table S2, Fig. S1). 6 days after seeding, about 60% of the cells were fully embedded in the gel (Fig. S2). For partially embedded cells, we define the “Degree of Invasion” (DoI) as the height difference between the collagen and apical cell surfaces (determined from the contact points of the force-indentation curves) divided by the cell height and subtracted from one (Fig. 4A). Collagen concentrations ranged 2.44−4.88 mg∙ml^−1^, and the apparent Young’s modulus of the pericellular collagen (E_col._) ranged 0.1−6 kPa (Fig. S3). In the data set, neither the cells’ Young’s modulus nor their degree of invasion (DoI) at times t ≤24 h differed significantly from those at times t ≥49 h (Fig. S4) when binned irrespective to collagen stiffness, and there was significant overlap in the distributions of pericellular collagen stiffness from different concentrations (Fig. S3), so data from all times and concentrations were pooled. We observed significant correlation between pericellular collagen stiffness and DoI. Invasion was enhanced for soft gels (E < 1 kPa) (Fig. 4B).

To determine the Young’s moduli of cells on top of or partially embedded within collagen I hydrogels, we used the sphero-conical model with substrate corrections as described above. In our experiments, the indenter shape, force and indentation depth are known, and the collagen and cell are assumed to be incompressible (*ν*_*1*_ = *ν*_*2*_ = 0.5)^51^,^52^. We discern the top layer thickness *h* (i.e. cell height) from the axial confocal fluorescence micrograph scanned before each indentation. We then assume that *E*_*2*_(the Young’s modulus of the gel underneath the cell) is equal to the average apparent Young’s modulus found from indenting the gel beside the cell (see Materials and Methods section). While this assumption may not always hold, our measurements directly beside the cell are more accurate than a bulk average, considering the inherent spatial heterogeneities of collagen gels. Using the method described above, we calculated the Young’s moduli of the cells.

Figure 4C shows the Young’s moduli of partially embedded cells as a function of the invasion depth, binned into three similarly populated groups. Cells 0−50% embedded (DoI < 0.50) had a median (±median absolute deviation) Young’s modulus of 0.74 ± 0.29 kPa, similar to the observed moduli of cells on glass (*p* = 0.61). Cells 50−75% embedded (0.50 < DoI < 0.75) had a median Young’s modulus of 0.99 ± 0.38 kPa, significantly stiffer (34%, *p* = 0.018) than cells 0−50% embedded. Cells 75−100% embedded (DoI > 0.75) had a median Young’s modulus of 1.36 ± 0.50 kPa, significantly stiffer again (35%, *p* = 0.037) than cells 50−75% embedded.

**Figure 5:**
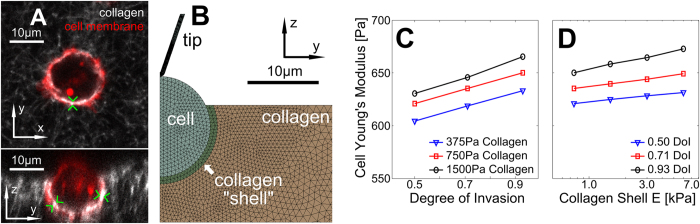
Influence of the surrounding substrate to cell stiffening during invasion. (**A**) Example of XY and YZ confocal fluorescence images of a partially embedded cell. Green arrows indicate high fluorescence intensity around the cell indicating collagen remodeling. (**B**) Finite element model of a partially embedded cell with a collagen “shell” for collagen displaced by the cell (here, R = 695 nm, θ = 18.8°, DoI = 0.93, cell diameter = 14 μm, E_cell_ = 0.75 kPa). For homogeneous substrates, E_shell_ = E_collagen_. (**C**) Corrected cell Young’s modulus versus degree of invasion for different collagen Young’s moduli for a homogeneous substrate; 6% apparent stiffening is observed between DoI = 0.5 and DoI = 0.93. (**D**) Cell apparent Young’s modulus versus collagen shell stiffness (collagen shell thickness = 1 μm, E_cell_ = E_collagen_ = 0.75 kPa, no correction is used); at most 3% apparent stiffening is observed for the parameters used.

In order to test if the observed stiffening is related to a phenotypic change or induced by the support of the surrounding material, we used finite element analysis to simulate indentations of round elastic cells embedded in elastic gels. We observed in some cells increased collagen fluorescence intensity at the cell-matrix interface, suggestive of ECM remodeling, perhaps by displacement and compression (Fig. 5A). In case such compression would result in local matrix stiffening, we incorporated thin stiff pericellular shells into the FE models in order to determine possible effects on the mechanical response. Figure 5B shows the FE model of a round elastic cell embedded in an elastic gel with an additional collagen shell used to simulate indentations. To quantify any mechanical response due to the buttressing effect of embedding, we virtually indented a cell (E_cell_ = 0.75 kPa) embedded at different depths (DoI ranging 0.5–0.9) in gels (with no shell) of various stiffness (E_gel_ ranging 375–1500 Pa) and assessed differences in the stiffness. The Young’s moduli resulting from fitting the virtual force-indentation curve show a marginal dependence on DoI, with an increase in stiffness of ~6% for DoI increasing from 0.50 to 0.93 (Fig. 5C). In the case of an additional denser collagen shell surrounding the cell up to 8-fold stiffer compared to the surrounding gel, we found an additional slight stiffening of ~3% for E_shell_ ranging 0.75–6.00 kPa (Fig. 5D). The amount of stiffening due to varying these effects is much less than the stiffening we observed experimentally during invasion, and well within cantilever calibration errors^53^.

**Figure 6:**
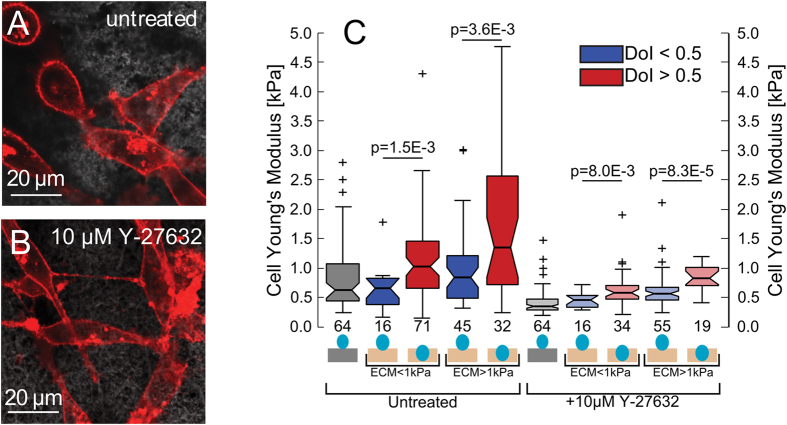
Young’s moduli of partially embedded cells is ROCK dependent. (**A,B**) Lateral confocal fluorescence images of cells (**A**) untreated and (**B**) treated with 10 μM Y-27632. (**C**) Box plots of Young’s moduli of cells (number of replicates are shown underneath) of untreated and treated with 10 μM Y-27632 on glass and partially embedded in collagen, binned by both DoI and pericellular collagen stiffness. All Young’s moduli are determined by fitting the indentation from 0.25−2.5 μm and corrected to account for the influence of the collagen substrate. *p*-values are calculated using the Mann-Whitney U test.

ANOVA testing revealed the two strongest predictors for cell stiffness were the degree of invasion and the local collagen stiffness, compared with the time after seeding and cell heights. We then binned the data by both the cells’ invasion depth and local collagen stiffness (Fig. 6C). On soft collagen (E_col._ < 1 kPa), cells 50−100% embedded (DoI > 0.5) were significantly stiffer (58% increase in median, *p* = 1.5E-3, all values are shown in Table 1) than cells 0−50% embedded (DoI < 0.5). On stiff collagen (E_col._ > 1 kPa), cells 50−100% embedded were 60% stiffer (*p* = 3.6E-3) than cells 0−50% embedded. For both DoI ranges, the cell and gel stiffness correlate.

### Invasion-associated stiffening is Rho/ROCK-dependent

Because the least-embedded cells had Young’s moduli similar to those measured on glass, and the stiffest cells were those invading the stiffer collagen regions, we hypothesized that more invasive cells are stiffer due to enhanced actomyosin contractility, which is associated with decreased F-actin depolymerization and increased actin fiber bundling—both factors which would cause increased cytoskeletal rigidity. Additionally, invasion of MDA-MB-231 cells in collagen and Matrigel has been shown to depend on the Rho/ROCK pathway, which regulates actomyosin contractility and amoeboid motility^28^,^30^,^54^,^55^.

We then repeated the measurements on cells in presence of 10 μM Y-27632 ROCK inhibitor. Treated cells adopted a characteristic spindle-like morphology (Fig. 6B, *c.f.* Fig. 6A). Compared to untreated cells, ROCK inhibition significantly reduced the Young’s moduli of cells on glass (51%, *p* = 2.39 E-7); cells embedded 0– 50% on soft collagen (31%, *p* = 0.10) and stiff collagen (33%, *p* = 3.6E-3); and cells embedded 50–100% on soft collagen (43%, *p* = 2.4E-5) and stiff collagen (39%, *p* = 0.017) (Fig. 5C). ROCK inhibition also diminished the increase in cell stiffness observed with increasing embeddedness. On soft collagen, 50–100% embedded cells were only 30% stiffer (*c.f.* 58%) than 0–50% embedded cells (*p* = 8.0E-3), and on stiff collagen, 50–100% embedded cells were only 47% (*c.f.* 60%) stiffer than 0–50% embedded cells (*p* = 8.3E-5). Additionally, 50–100% embedded cells on stiff collagen were 42% stiffer (*c.f.* 31%) than those on soft collagen (*p* = 1.1E-3). This confirms that ROCK-mediated contractility plays a significant role in the invasion-associated cell stiffening we observed.

### Fully embedded cells exhibit similar invasion-associated stiffening

**Figure 7:**
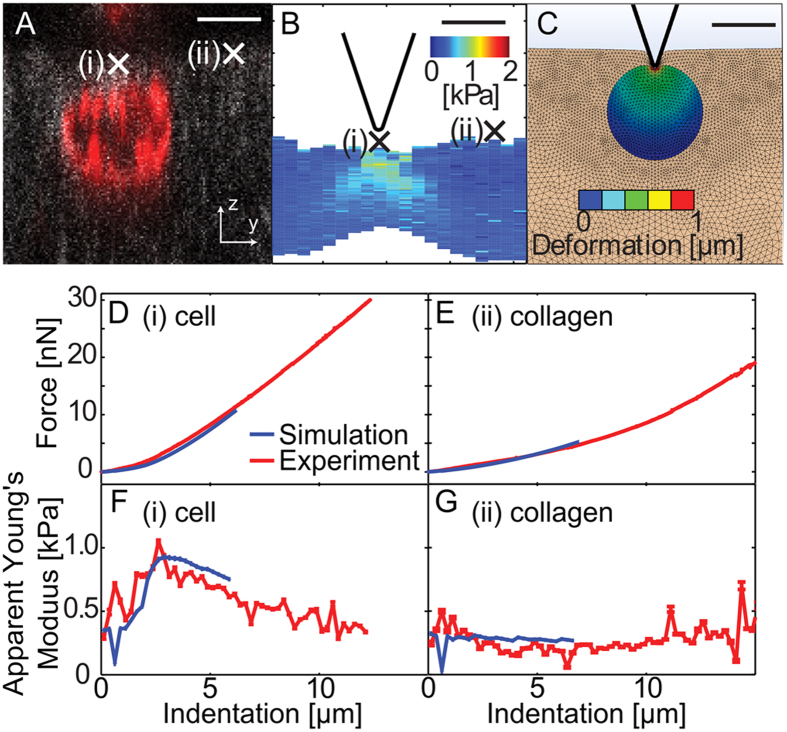
Determination of Young’s moduli of MDA-MB-231 cells fully embedded in collagen I hydrogels. (**A**) Axial confocal fluorescence micrograph of a cell that has fully invaded into the collagen matrix to a depth of ~2 μm, taken using the same protocol as in Fig. 3. (**B**) Spatially co-registered elasticity map, showing probe size. (**C**) Axisymmetric finite element simulation of a fully embedded cell being indented; the color scale represents the total deformation of the cell with the tip indenting 3 μm into the sample. Scale bars: 10 μm. (**D,E**) Experimental (red) and simulated (blue) force-indentation curves from points (i) and (ii) taken over the cell (**D**) and gel (**E**). (**F,G**) Apparent Young’s moduli calculated from the experimental (red) and simulated (blue) force-indentation curves in (**D,E**).

We extended our methods to allow accurate determination of fully embedded cells. Fig. 7A,B show the axial confocal image and elasticity map of the apparent Young’s modulus of an MDA-MB-231 cell embedded in collagen. Figure 7C shows a finite element model of this cell, in which indentations were simulated (at points corresponding to (i) and (ii) in Fig. 7A,B). The cell’s position and imposed modulus were iteratively adjusted in the simulation until the simulated force-indentation curves (Fig. 7D,E in blue) resulted in apparent Young’s moduli (Fig. 7F,G, blue) that differed minimally from the apparent Young’s moduli fitted from the experimental force-indentation curves (Fig. 7D–G, red). Figure 7 shows that the optimal choice of imposed parameters in simple finite element models can yield virtual force-indentation curves that match the experimental force-indentation curves remarkably well, along with the depth-dependent apparent Young’s moduli resulting from fitting them to the sphero-conical model. While many fully embedded cells were measured experimentally (n = 64), only those at invasion depths ≤3.5 μm could be quantified with this method despite the AFM probe reaching the depth of the cell because more deeply embedded cells have lower mechanical contrast (Fig. S5). 13 cells were analyzed with this approach; with 3 stiff (>10 kPa) outliers omitted, the median Young’s modulus was 1.5 ± 0.4 kPa (Fig. S6). This is ~60% stiffer than non-embedded cells and consistent with the statistically robust trend observed among partially embedded cells.

## Discussion

It is well accepted that cells feel and adjust to mechanical properties of their environment^56^. For fibroblasts on 2D surfaces, the dependence of cell stiffness and substrate stiffness has been studied systematically over a large substrate stiffness range^57^. For fibronectin-coated polyacrylamide gels with elastic moduli below 20 kPa, the stiffness of fibroblasts were equal or slightly lower than the substrate. We observe in 3D a similar behavior; for comparable depths of invasion, the gel and cell stiffness correlate.

Cell stiffening can be viewed in the general framework of tensegrity models developed by Ingber *et al.*^58^. For a simple active cytoskeletal model consisting only of myosin II, actin filaments, and cross-linkers, motor protein induced stiffening has been observed^59^. Furthermore, a linear dependence between the cytoskeleton contractile prestress and the shear modulus has been observed for cells adhered to a surface^60^,^61^. Such a linear dependence if applied to our data would correspond to an 80% change in actin-myosin activity during the invasion of MDA-MB-231 cells into collagen I matrixes. By the same reasoning, our data indicate treatment with 10 μM Y-27632 ROCK inhibitor would diminish actin-myosin activity by twofold. An additional mechanism for cell stiffening during invasion could be the formation of F-actin rich uropod-like structures at the cell rear during rounded-cell vertical migration into 3D ECM. Poincloux *et al.* reported this effect for MDA-MB-231 cells invading into Matrigel^30^. Our observations corroborate the rounded-cell invasion model. However, MDA-MB-231 cells are capable of exhibiting motility across the spectrum of amoeboid and mesenchymal modes, as well as collective cell migration^62^. We observed unembedded and invading cells with rounded and elongated morphologies, and with invadopods and pseudopods (Fig. S7). The nucleus plays an important role in cell motility as well^22^,^63^. The movement of a cell past adjacent cells in epithelial sheets has been observed to be rate-limited by the passage of its nucleus past those of its neighbors^16^. In addition, for cells in which the nucleus separates the cell into leading and trailing compartments, lobopodia formation may be due to increased hydrostatic pressure caused by forward displacement of the nucleus^64^. Future studies are needed to determine any contribution to the stiffening we observe of the actin cap involved in such nuclear displacements.

It is important to acknowledge that while both the cell and its microenvironment are extremely complex discrete structures, the models used and developed here all take a continuum mechanics approach. The assumptions for two-layer substrates made by Dhaliwal and Rau^50^ are nearly identical to the assumptions made by Sneddon^38^ (linear elasticity, flat infinite sample, small deformation, isotropic, non-adhesive contact) with the only exception being an additional boundary condition with a bonded substrate of different elastic properties. When the integral term in Eq. (17) is zero due to *μ* = 1 or *h* → ∞, the problem is identically reduced to that considered by Sneddon^38^. Thus, the model presented here will only be valid in small strain regimes and errors arise when 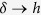, especially in the case of *E*_*2*_ > *E*_*1*_. This approach may also be used in the case of samples with finite thickness and a rigid substrate (*E*_*2*_ >> *E*_*1*_). The results are then similar to the solution shown by Dimitriadis *et al.*^65^ when a parabaloid indenter is assumed, but differ from the solution given by Gavara *et al.*^66^ for a conical indenter (data not shown).

We used axially symmetric finite element models to verify invasion-associated stiffening in cells that were fully embedded in the collagen. Finite element models assume a simple elastic behavior, and do not reflect remodeled or spatially heterogeneous collagen, non-spherical cells, and general experimental noise. While small deviations are thus not unexpected, we observe good agreement between the experimental and simulated data in this work. To further investigate the suitability of a continuum approximation, we conducted additional finite element simulations of indentations of elastic materials with element mesh sizes similar to or less than the probe radius show negligible artifacts (Fig. S8). On average and in the length scale of our measurements, the apparent Young’s modulus of the collagen gels was independent of depth when calculated by the sphero-conical model (Fig. S9). Near some cells we observed a stiffer upper layer of the collagen gel in the AFM signal and increased fluorescence intensity (Fig. S9).

We have demonstrated an approach combining AFM, confocal microscopy, and simulation that allows for the extraction of quantitative data from indentations on submerged objects. Despite the cells’ being embedded in ECM, the force responses of the cell and ECM can be successfully decoupled to achieve single cell force resolution in a 3D microenvironment. We observed that the cells stiffen significantly as they invade into collagen I matrices.This stiffening depends on Rho/ROCK-dependent actomyosin contractility, and cannot be explained as a consequence of buttressing support provided by the pericellular matrix as demonstrated by the finite element models. Invasion was enhanced on softer gels, which may reflect either that invading cells soften the matrix by active rearrangement or proteolysis of adjacent fibers by invasive cells, or that invasion is more likely in intrinsically more compliant regions. Cell stiffness was also correlated to matrix stiffness, possibly indicative of force balance and mechanical equilibration. These novel observations illustrate the importance of the 3D microenvironment to understanding the mechanics and behavior of cells in their physiological context. The methods presented here enhance the potential of AFM for use in cell mechanics assays to identify cells’ response to specific treatment or growth conditions while cultured in ECM-like materials.

## Materials and Methods

### Piecewise depth-dependent fitting method for force-indentation curves

To fit the force-indentation (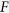-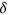) data and approximate the apparent Young’s Modulus (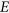), a segmented regression approach is used in which the 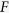-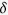 data is binned into intervals of indentation (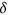), the curve segment is linearized, and 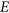 is extracted from the slope of the linearized data^67^. This type of analysis provides two key benefits: errors from the contact point are reduced^18^,^40^, and the depth-dependence of the fitting provides the ability to extract quantitative information regarding mechanical heterogeneity. We consider changes in the force response for an axisymmetric indentation on an infinite, elastic half-space:


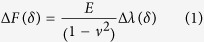


where *λ* is a function that describes the indenter geometry. *λ* is a power law function for parabolic and conical indenters^68^ and may otherwise be approximated as a power law function for small piecewise segments of the data:


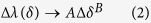


The sphero-conical tip geometry (Eq. 15, 16) does not explicitly obey an exact power law, but follows one approximately. Therefore the power law fit parameters *A* and *B* are approximated locally for a given indentation depth using least-squares regression (for example in SI units, when R = 695E-9, θ = 18.8°, δ ranges from 0.25E-6 to 2.50E-6, then A = 1.289E-4, B = 1.357 with r^2^ = 0.9996). Once the power-law dependence is known, the 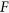-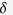
segment may then be linearized by:


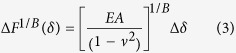


The slope of 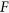 may be taken with respect to 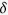, which will produce a constant value (the intercept from the linear fit is discarded):


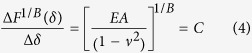


Therefore, the apparent Young’s modulus from fitting may be estimated as


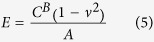


The size of the indentation bin may be adjusted depending on the desired resolution from the analysis of the force curve. For example, to increase sensitivity to local sample heterogeneity, smaller indentation bins (e.g. 250 nm) may be used. To approximate a Young’s modulus which is coarse-grained over a larger volume of the sample, a higher (e.g. 2.25 μm) bin size may be used. When using the sphero-conical model, for tip radii *R* >> *δ*, the resulting values for *A* and *B* are identical to the Hertz model (*B* = 1.5), and for *R* → 0, the resulting values for *A* and *B* are identical to the Sneddon model (*B* = 2.0).

### Derivation of the contact model for a sphero-conical probe indenting an elastic half-space

Following Sneddon’s method, we derive a non-adhesive elastic contact model for a conical indenter with a spherical tip that features continuous curvature at the transition point^38^, as illustrated in Fig. 1A,B. The integral equations are solved using Mathematica 8 (Wolfram Research, Illinois, USA).

Eqs. 6 and 7 together give a piecewise function describing in cylindrical coordinates the axisymmetric surface of the sphero-conical tip, where 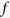 is the axial coordinate originating at the tip and 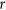 is the radial coordinate. They are normalized to the contact radius 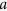 (such that 0 ≤ *r* ≤ 1), and are given by:


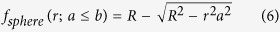



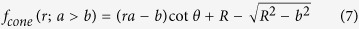


where


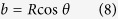


is the transition point between the cone and sphere, and the function 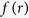 and its first derivative 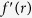 are continuous at the transition point 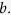


To calculate the indentation depth 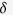
and applied force 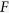, the Abel integral 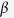
is solved:


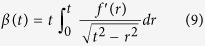


where 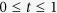. Because of the nature of the transition from the spherical apex to the cone angle, 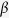 must be solved in two separate regimes for 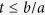 and 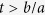:


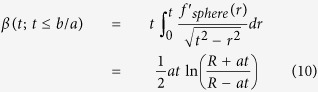



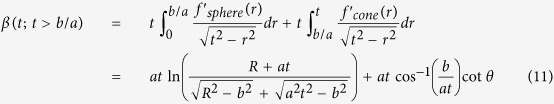


The indentation depth of the tip is given by satisfying the boundary condition 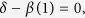 thus in the spherical region:


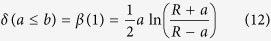


And in the conical region:


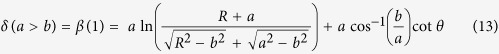


The applied pressure may be integrated for using the formula:


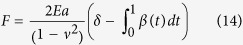



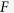
is separated the same way as 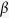. In the spherical region:


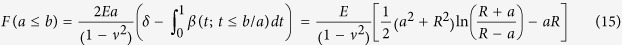


And in the conical region:


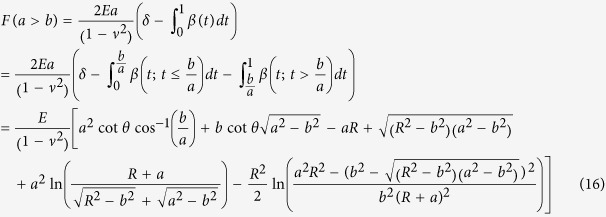


Thus, the applied force 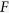 by a sphero-conical tip at indentation depth 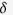
may be determined by numerically solving Eqs. (12), (13), (15), and (16). To do this, the MATLAB built-in function fzero is used to determine the value 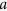 from Eqs. (12) and (13), which is then inserted into Eqs. (15) and (16). In the context of the depth-dependent fitting method used in this work and described in Eqs. (1)–(5), 18, 21, 24, 25, 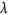 is equal to the terms enclosed in square brackets in Eqs. (15) and (16).

### Analytical model of indentation of the bonded two-layer linear elastic material

Dhaliwal and Rau presented a generalized analytical solution to the indentation problem of two bonded layers in the form of a Fredholm Integral Equation of the Second Kind^40^,^50^, where *h* is the height of the first layer, 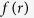
is the function describing the axisymmetric tip shape, *a* is the contact radius, *E*_1_ and *ν*_1_ are the Young’s modulus and Poisson ratio of the top layer, and *E*_2_ and *ν*_2_ are similar quantities for the bottom layer (Fig. 3A):


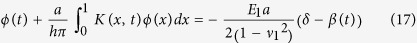



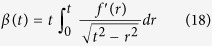



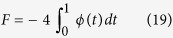



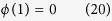


with


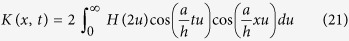



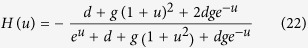



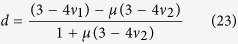



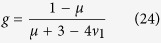



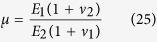


The function φ is related to the normal pressure distribution of the indentation which depends on the indenter geometry. The above equations are solved numerically in MATLAB using the program Fie employing theNyström method^69^ for Eq. (17), Eq. (18) is solved analytically, the built-in function fzero for Eq. (20), and the built-in functions quadv and trapz for all integrals to produce a force-indentation relationship and determine the effects of the bottom layer in determining the Young’s modulus of the top layer.

### Collagen preparation

Bovine collagen I (Nutragen, Advanced Biomatrix) gels were attached to glass surfaces using a procedure similar to Pelham and Wang^70^. Glass-bottom dishes were ultrasonicated in ethanol for 10 min, then ultrasonicated in ultrapure water for 10 min. Dishes were blown dry with N_2_ gas, plasma cleaned in O_2_ gas for 5 min, incubated in 1% (3-Aminopropyl)triethoxysilane in ethanol for 30 min, then washed with ethanol and ultrapure water. Surfaces were then incubated in 0.5% glutaraldehyde in 1 × DPBS for 1 h, washed with ultrapure water, then allowed to dry vertically. Purified bovine collagen I (6.1 mg∙ml^−1^) was mixed on ice with 10 × DPBS, 0.1 M NaOH, and ultrapure water at a ratio of 32:4:3:1 to form a 4.88 mg∙ml^−1^ gel. The collagen was diluted with ultrapure water and the amount of NaOH was linearly decreased to form gels at lower concentrations. 200 μL was pipetted onto functionalized dishes and spread with the pipette tip. Dishes were then incubated 1.5 h at 37 °C, 5% CO_2_, after which the gels were gently covered with 37 °C 1 × Dulbecco’s phosphate buffered saline (DPBS). Collagen was stained with 0.02 mg∙ml^−1^ Atto 465 NHS for 30 minutes, which was suspended at 2 mg∙ml^−1^ in dimethyl sulfoxide (DMSO).

To quantify the gels’ pore size distributions, four collagen hydrogels were prepared as described above, but were not stained with fluorescent dyes. After 24 h, the DPBS was removed from the gels and exchanged with growth media (to two of the dishes, 50 μl cell suspension was also added; one of the gels seeded with cells was also supplemented with 10 μM Y-27632) and incubated 96 h. Gels were then rinsed with DPBS and fixed with 3.7% paraformaldehyde in DPBS for 0.5 h (one of the two unseeded gels was left unfixed). The gels were then rinsed again in DPBS, covered in DPBS, and stored at 4 °C for 24 h. The gels were then imaged by confocal reflection microscopy with circularly polarized light. Samples were imaged by a 100X oil objective with 640 nm laser excitation of enough intensity to transmit reflected light through the dichroic mirror with sufficiently high signal:noise ratio. On each sample, ~20 10 μm × 10 μm oversampled scans (39 nm/pixel) were recorded. Resulting intensity bitmaps were imported as stacks and binarized in ImageJ^71^ (Image >Adjust >Auto-Threshold [Mean; White objects on Black Background; Stack; Use Stack Histogram). Binarized images were then segmented (Process >Binary >Watershed). The pores were then analyzed (Analyze >Analyze Particles … [10-Infinity; 0.00–1.00; Pixel Units; Show Ellipses; Exclude on edges; Include holes]). The pore size of each ellipse was taken as the average length of its minor and major axes. The areal porosity was also calculated in ImageJ (Invert >Analyze >Measure >%Area).

### Cell culture

MDA-MB-231 metastatic breast cancer cells from ATCC were cultured at 37 °C and 5% CO_2_ in 1 × Dulbecco’s Modified Eagle Medium containing 4.5 mg∙ml^−1^ D-glucose and L-glutamine supplemented with 10% Fetal Bovine Serum. Cells were cultured as in^15^ using Cellstripper™ in place of trypsin. For experiments with inhibitors, growth medium was supplemented with 10 μM Y-27632 at the time the cells were seeded on the dishes. Cells were seeded on top of polymerized collagen gels or glass-bottom dishes 6−96 hours prior to measurements in growth medium for use in experiments. Cell membranes were stained using 2.5 μg∙ml^−1^ CellMask™ Deep Red Plasma membrane stain for 30 minutes, which was suspended at 1 mg∙ml^−1^ in DMSO. Cell measurements and staining were performed at 37 °C in 1 × Hank’s Balanced Salt Solution (HBSS) containing calcium and magnesium. For experiments with inhibitors, HBSS was supplemented with 10 μM Y-27632.

### Atomic force microscopy and confocal fluorescence microscopy

The AFM and confocal fluorescence measurements were performed on a combined system consisting of an Asylum Research MFP-3D-BIO AFM and a Picoquant Microtime 200 confocal laser scanning microscope^18^,^49^. Team NanoTec LRCH-750 AFM probes were used. Spring constants were determined using the thermal energy dissipation method (typically ~0.15 N∙m^−1^)^72^,^73^. The AFM tip was aligned in the confocal volume and confocal fluorescence images were scanned in lateral and axial planes. Data collected for elasticity maps was recorded by taking a line measurement of 40 μm with 20 force-indentation measurements in the axial plane, normal to the cantilever (one indentation per 2 μm) with 3 μm∙s^−1^ approach and retraction speeds in the elastic plateau region (MDA-MB-231 cells^17^ and collagen^74^ have low loss tangents at 0.1–1 Hz) and a variable trigger force (typically 15−35 nN). Because we do not fit the indentation curves globally, the depth-dependent apparent Young’s moduli we determine from piecewise fitting are explicitly independent of the choice of the trigger force. The choice of trigger force is made only to control the total indentation depth, which depends on the sample stiffness. For cells plated on glass, each cell is indented 4 times in force-volume mode over a 4 μm^2^ area in the central nuclear region.

### Equipment and settings

Each confocal scan was 60 × 60 μm, 256 × 256 pixels (234 nm/pixel). Each scan took ~1 min. Two pulsed diode lasers (ex: 470 nm, 640 nm) were used to excite the fluorophores (Atto 465-NHS for collagen and CellMask Deep Red HCS for cell membranes). A water immersion objective (Olympus LUMFL60X, 60 × magnification, 1.1 NA, 1.5 mm W.D.) was used. A dual bandpass dichroic (Chroma 467/638rpc) was used to separate laser light and fluorescent light and a second dichroic (Chroma 600dcxr) is used to separate the green and red fluorescence light, which are each passed through an emission filter (Chroma HQ690/70m; Semrock FF01-520/35) and collected by a single photon sensitive avalanche photodiode (Picoquant PDM series). For each detection channel an intensity micrograph is recorded and constructed in the operating software (Picoquant SymphoTime), exported as a 16 bit bitmap, and merged in ImageJ to create false color images. Annotations were added to the images thereafter in Adobe Illustrator or Inkscape.

The scanning electron microscope image in Fig. 1A was taken with an XL30 ESEM-FEG at the LeRoy Eying Center (5 kV, 3500×).

### Analytical models and finite element analysis

We used Mathematica 8 (Wolfram Research, Illinois, USA) to solve the integral equations for the sphero-conical and two-layer contact models. Finite element analysis was performed using ANSYS Workbench 14.0. The models were axially symmetric around the center of the tip and cell to increase computational efficiency. The collagen was modeled as an Ogden 1^st^ order solid (α_1_ = 2, identical to Neo-Hookean solid) with height and radius of 100 μm with a fixed support on the bottom boundary. The cell was modeled as a spherical inclusion bonded to the collagen with a different Young’s modulus but otherwise similar material properties. The AFM tip was modeled as a sphero-conical tip with dimensions similar to those used in the experiment and Young’s modulus on the order of GPa. The tip had a triangular mesh size of 50 nm and the contact between the tip and sample was assumed to be frictionless. The cell and collagen mesh had an element length of 500 nm within 20 μm from the tip, which then tapered up to 1 μm. All elements had midside nodes. Indentations between 4 μm and 7 μm were performed in 5 nm incremental steps depending on when the simulation failed to converge due to numerical instability occuring at large deformations. The Poisson ratios of the cell and the collagen were set to 0.45.

### Data analysis

Data analysis was performed using MATLAB. Mann-Whitney U testing was performed with the built-in ranksum command in MATLAB (two-sided, approximate for large n). Boxplots were produced using the MATLAB built-in function with whisker lengths 1.5 times the interquartile range. ANOVA testing was performed using MATLAB function anovan. Cells and collagen were assumed to be incompressible at the length scale of AFM indentation (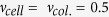)^51^,^52^, however experiments performed on both bulk collagen^75^ and single ECM fibrils^76^ demonstrate *ν*_*col.*_ > 0.5, presumably due to water flux, thus some systematic errors may arise from this choice. AFM force-indentation curves are fit assuming a sphero-conical tip geometry. Unless otherwise noted, the indentation depths used for fitting is fixed to 0.25–2.50 μm.

To determine the Young’s modulus of partially embedded cells, the two-layer deconvolution technique is applied. The average pericellular collagen (bottom layer) Young’s modulus (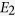) is calculated for each cell (from 3 curves and distance 4−12 μm from the cell on each side if available) and the cell (top layer) height 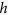 is estimated from the axial confocal fluorescence micrograph recorded before the indentations. The average apparent Young’s modulus of the cell is determined from a set of 3–4 experimental force-indentation curves from the highest part of the cell’s apical surface. The cell Young’s modulus is then corrected for the collagen substrate effect (shown in Fig. 3B) iteratively until convergence is reached. To determine the Young’s modulus of cells on glass, the force-indentation data is fit and averaged over 4 curves per cell.

To determine the Young’s modulus of fully embedded cells, one experimental force-indentation curve over the highest part of the cell’s central region is used to represent the cell. The curve over the cell is fit using Eqs. (1, 2, 3, 4, 5) piecewise in 250 nm intervals to find the depth-dependent apparent Young’s modulus. Finite element models are generated, the invasion depth and diameter of the cell is estimated from the axial confocal micrograph, and the Young’s modulus of the collagen is determined in the same way as partially embedded cells. The cell’s Young’s modulus is initially guessed, and an indentation by a rigid (~GPa) probe with geometry similar to that of the experimentally used AFM probe is then simulated using finite element analysis, yielding a simulated force-indentation curve. The simulated curve is fitted in an identical manner to the experimental curve and the results are compared. Simulations are repeated in subsequent models in which the depth of the cell is adjusted in 0.25 μm increments, and the Young’s modulus of the cell is adjusted in 0.1 kPa increments, until the experimental and simulated depth-dependent apparent Young’s moduli differ minimally.

## Additional Information

**How to cite this article**: Staunton, J.R. *et al.* Correlating confocal microscopy and atomic force indentation reveals metastatic cancer cells stiffen during invasion into collagen I matrices. *Sci. Rep.* **6**, 19686; doi: 10.1038/srep19686 (2016).

## Supplementary Material

Supplementary Information

## Figures and Tables

**Table 1:** Young’s moduli of MDA-MB-231 cells for different invasion depths, collagen stiffnesses and treatments.

	Substrate	Degree of Invasion (DoI)	Number of cells	Young’s Modulus of the Cell [kPa]	
				(mean ± s.e.m.)	(median ± m.a.d.)
Untreated	Glass	0	64	0.86 ± 0.8	0.63 ± 0.27
	Col E <1 kPa	<0.5	16	0.65 ± 0.10	0.65 ± 0.29
	Col E >1 kPa	<0.5	45	0.97 ± 0.09	0.84 ± 0.37
	Col E <1 kPa	>0.5	71	1.14 ± 0.08	1.03 ± 0.42
	Col E >1 kPa	>0.5	32	1.71 ± 0.21	1.35 ± 0.68
	All	Fully Embedded	10	1.4 ± 0.2	1.5 ± 0.4
+10 μM Y-27632	Glass	0	64	0.43 ± 0.02	0.36 ± 0.08
	Col E <1 kPa	<0.5	16	0.45 ± 0.03	0.45 ± 0.10
	Col E >1 kPa	<0.5	55	0.62 ± 0.04	0.56 ± 0.11
	Col E <1 kPa	>0.5	34	0.64 ± 0.05	0.58 ± 0.12
	Col E >1 kPa	>0.5	19	0.84 ± 0.05	0.83 ± 0.17
