# Supplementary material for: Correlating confocal microscopy and atomic force indentation reveals metastatic cancer cells stiffen during invasion into collagen I matrices
Source: Sci Rep. 2016 Jan 27;6:19686. doi: 10.1038/srep19686 (PMC4728602; doi:10.1038/srep19686)
Supplement: Supplementary Information [file srep19686-s1.pdf]

Supporting Information

Correlating confocal microscopy and atomic force indentation reveals metastatic cancer cells stiffen during invasion into collagen I matrices

Jack R. Staunton, Bryant L. Doss, Stuart Lindsay, Robert Ros

Table S1: Comparison of different contact models

	Linearized Fit		Least Squares Fit	
	**0.25-2.50µm**	**1.00-5.00µm**	**0.25-2.50µm**	**1.00-5.00µm**
**Hertz**	-37.6%, 0.9964	-31.3%, 0.9992	-29.2%, 0.9941	-31.3%, 0.9994
**Sneddon**	+51.9%, 0.9145	+12.5%, 0.9408	+153.8%, 0.9381	+75.1%, 0.9612
**Sphero-conical**	-10.0%, 0.9986	+0.9%, 0.9988	-6.7%, 0.9994	-4.1%, 0.9997

Errors in the apparent calculated Young's moduli from the imposed simulation Young's moduli and r^2 values from fitting the finite element simulated curve using linearized (used in this work) and least squares routines, along different indentation intervals, and with different tip geometry models. There are no contact point errors due as it is exactly known. The simulated tip is sphero-conical with R=695nm, θ=18.8° and the mesh size of the sample is 500nm. The sample is very large (height, width 100µm) and is flat, homogeneous, elastic, and isotropic.

Table S2: Collagen pore size, pore area, and areal porosity.

	Pore Size [μm] (Mean ± std.)	Pore Area [μm^2] (Mean ± std.)	Areal Porosity [%] (Mean ± std.)
Fixed, MDA untreated	0.5 ± 0.3	0.3 ± 0.3	67 ± 7
Fixed, MDA 10 μM Y-27632	0.5 ± 0.3	0.3 ± 0.4	68 ± 12
Fixed, No cells	0.5 ± 0.3	0.3 ± 0.3	68 ± 10
Unfixed, No cells	0.6 ± 0.3	0.3 ± 0.4	72 ± 9

The gels were imaged by confocal reflection microscopy with circularly polarized light of 640 nm. On each sample, ~20 10 μm × 10 μm oversampled scans (39 nm/pixel) were recorded. Resulting intensity bitmaps were imported as stacks and binarized in ImageJ[1] (Image>Adjust>Auto-Threshold [Mean; White objects on Black Background; Stack; Use Stack Histogram). Binarized images were then segmented (Process>Binary>Watershed). The pores were then analyzed (Analyze>Analyze Particles… [10-Infinity; 0.00-1.00; Pixel Units; Show Ellipses; Exclude on edges; Include holes]). The pore size of each ellipse was taken as the average length of its minor and major axes. The areal porosity was also calculated in ImageJ (Invert>Analyze>Measure>%Area).

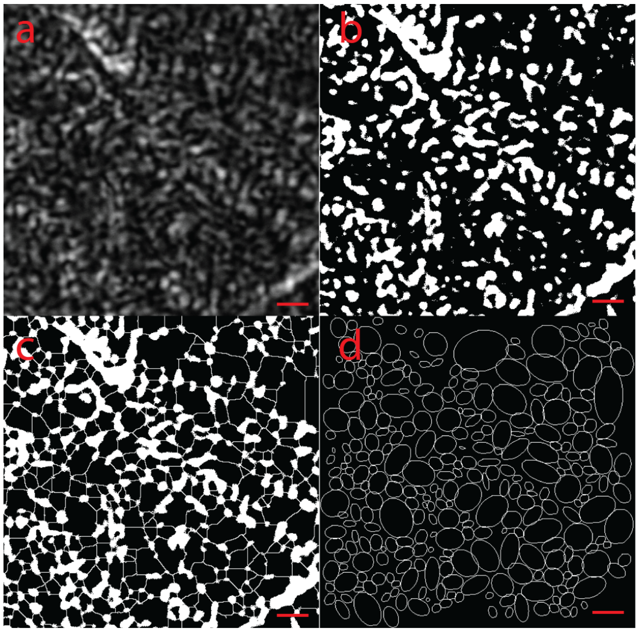

Fig. S1: Collagen pore size distribution. (a) Example confocal reflection micrograph of collagen I hydrogel. (b) Image in (a) after thresholding and binarization. (c) Image in (b) after Watershed segmentation. (d) Elliptical particle analysis of image in (c).

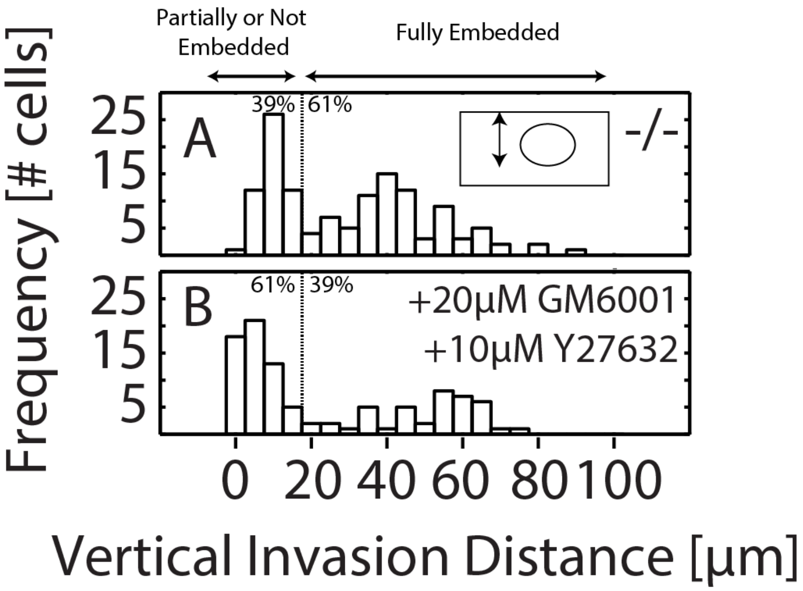

Fig. S2: Distribution of the vertical invasion distances of MDA-MB-231 cells 6 days after seeding on collagen I matrixes. (A) Untreated cells and (B) cells treated with MMP inhibitor GM-6001 and the ROCK inhibitor Y-27632.

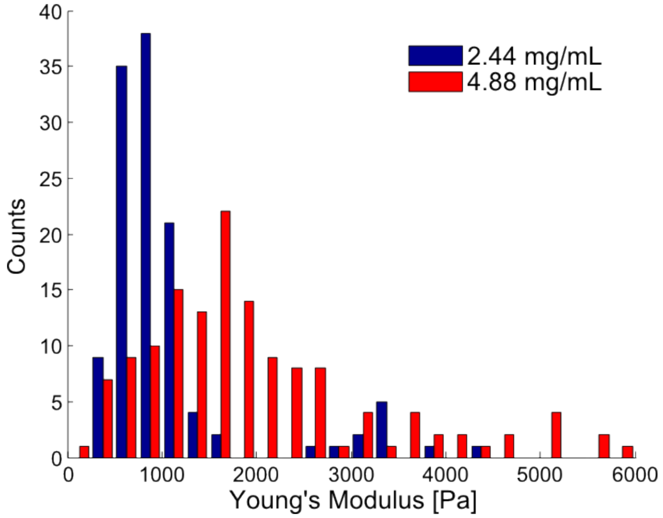

Fig. S3: Histograms of Young's moduli of pericellular bovine collagen I from hydrogels formed at different initial collagen concentrations.

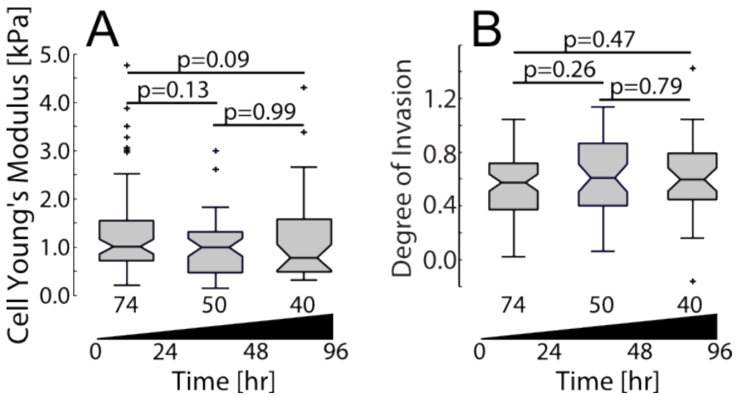

Fig. S4: Box plots showing (A) stiffness and (B) degree of invasion of partially invaded MDA-MB-231 seeded on collagen I matrixes at various time points. P-values are calculated from the Mann-Whitney U test.

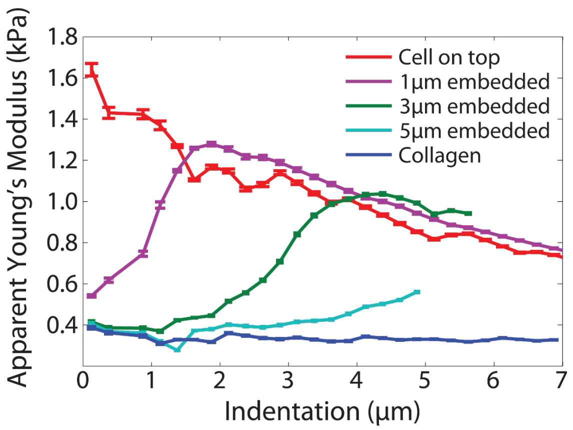

Fig. S5: Plot of the depth-dependent Young's moduli of fully embedded cells at various depths from finite element analysis simulations. The parameters used are a sphero-conical tip with apex radius 745 nm and half-angle 18.81°, the cell Young's modulus is 2.0 kPa and collagen Young's modulus is 0.3 kPa (both with Poisson ratio 0.45), and the radius of the cell is 6.5 µm. As the cell moves from sitting on top of the collagen gel with a protrusion of 1 µm (red) to being 5 µm embedded (teal), both the highest observed value of the Young's modulus and the rate at which the signal transitions from collagen to cell decrease. Thus, the mechanical contrast decreases as the cell becomes further embedded in the collagen, so the analysis of the fully embedded cells must be limited to cells that have not invaded too deeply. The bin from 500-750 nm was omitted to remove finite element meshing artifacts.

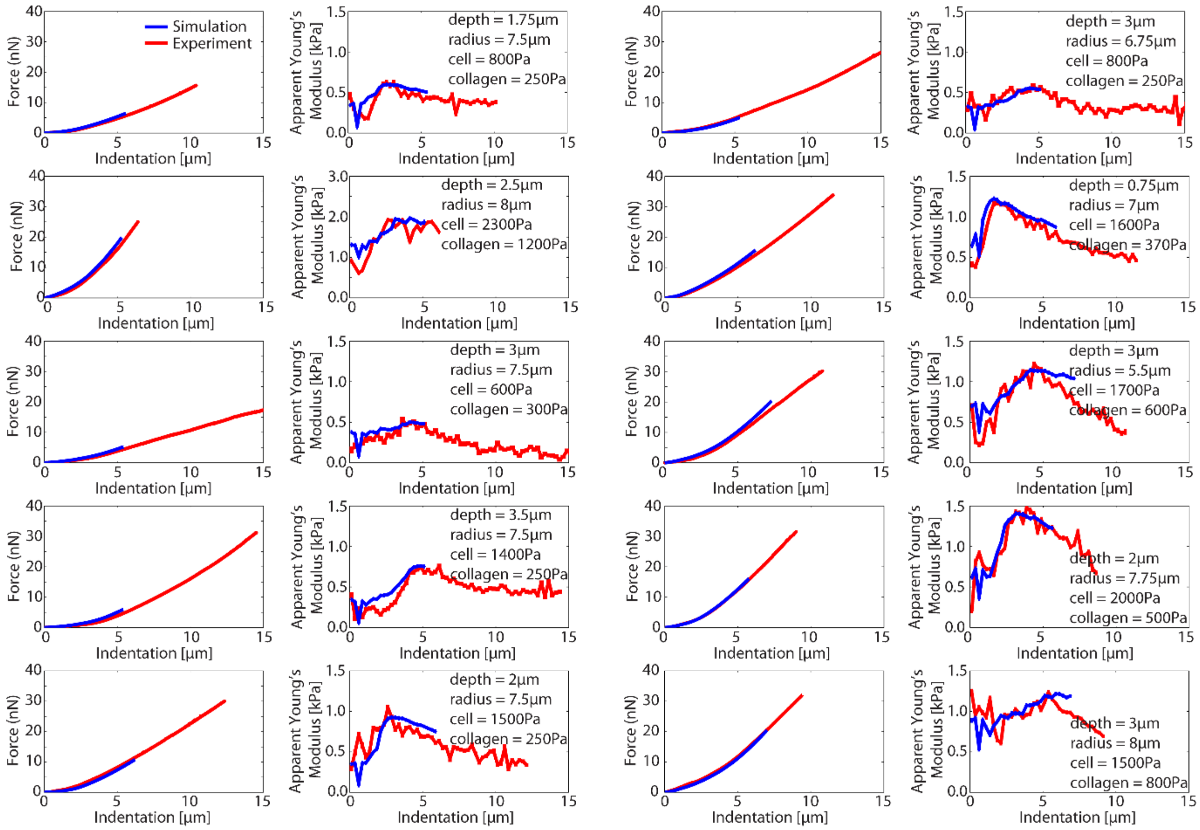

Fig. S6: Ten fully embedded cells with the experimental force-indentation curves and depth-dependent fits (red) overlayed with the finite element simulation results (blue) from ANSYS. For each cell, the physical dimensions and elasticity parameters of the sample are given. The tip is modeled to be sphero-conical with the same dimensions as used in the experiment. The mesh of the cell and collagen is triangular with element length of 500 nm for a radial distance of 20 µm from the top and center of the collagen, and then tapers to 1µm. The simulation is axisymmetric, and the collagen has a depth and radius of 100 µm. The mesh of the tip is triangular with element length of 50 nm. The contact point of the force-indentation data is determined manually for each of these examples. The bin size for the depth-dependent fits are 250 nm.

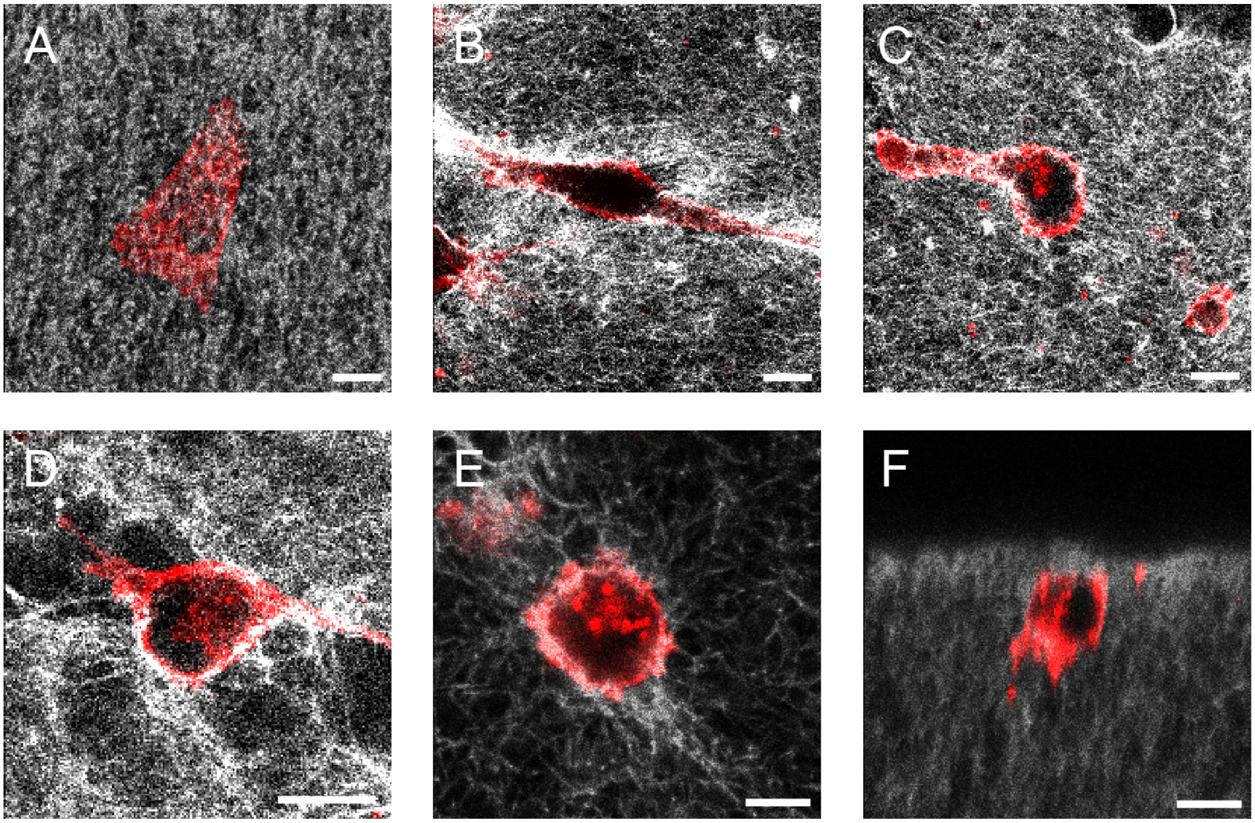

Fig. S7: Range of morphological features during invasion. A) Cell on top of gel with polygonal morphology. B) Invading cell with elongated, mesenchymal morphology. C) Partially embedded rounded cell with large podosome. D) Fully embedded rounded cell with long protrusions. E) Partially embedded rounded cell with blebs. F) Fully embedded cell with invadopods. All scale bars 10 µm.

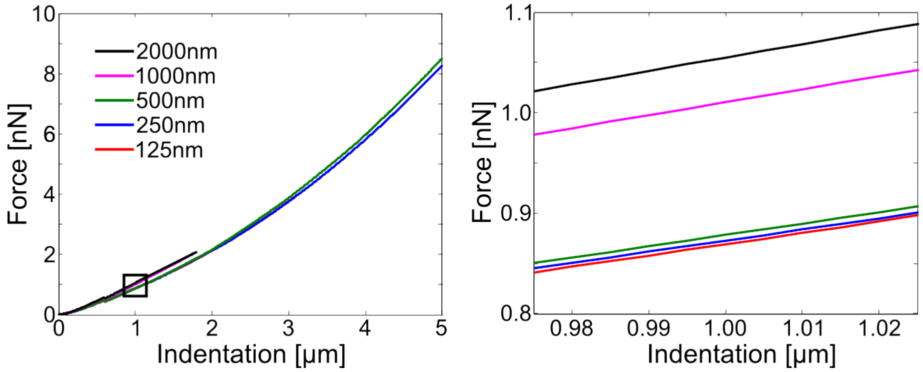

Fig. S8: Effect of the size of the sample mesh on the finite element simulated force-indentation curve. The simulated tip is sphero-conical with R=695nm, θ=18.8° with indicated mesh size. The sample is very large (height, width of 100µm) and is flat, homogeneous, elastic, and isotropic. (left) Zoom-out of the simulated curves, and (right) zoom-in of the simulated curves. Errors arise when the mesh size is larger than the probe radius, but are otherwise marginal.

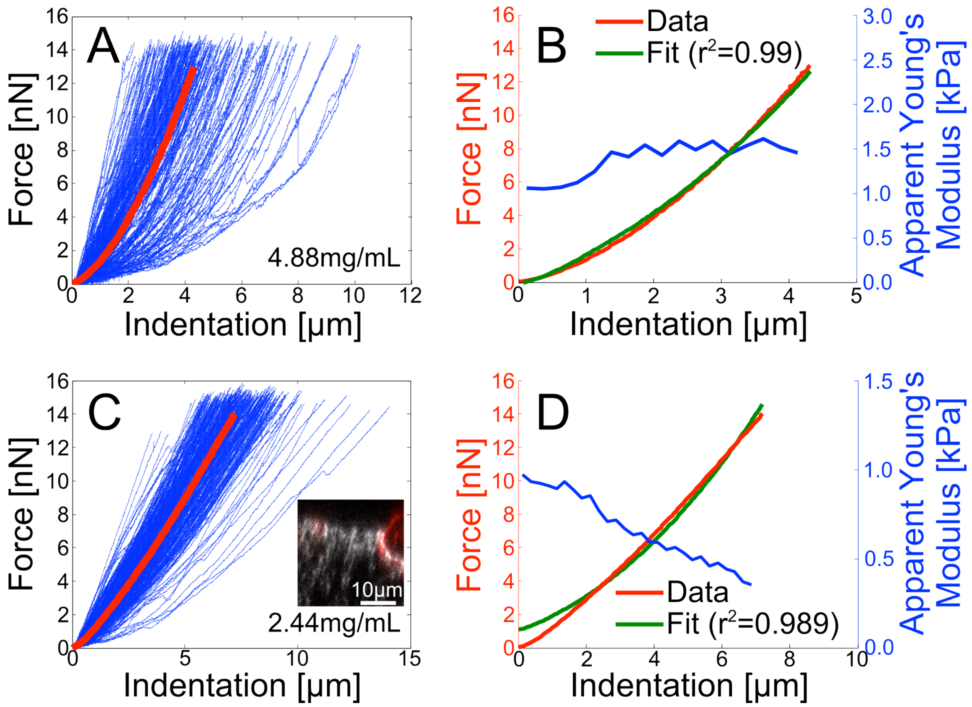

Fig. S9: Force-indentation values and fits of bovine collagen I. (A) (blue) Force-indentation curves on collagen (4.88 mg/mL) from an experiment aligned at the contact point, and (red) the average force-indentation curve using the mean indentation at 0.1 nN force intervals (method 2 from Sokolov et al.[2]. (B) The average force-indentation curve (red, left axis) from (A) fit using the methods in this paper along the entire curve (green, right axis) and the resulting Young's modulus shown from fitting along 250 nm indentation intervals (blue, right axis). (C, D) same as (A, B) but for 2.44 mg/mL collagen. Inset in (C) shows a YZ confocal image from 2.44 mg/mL collagen, showing some mechanical heterogeneity in the collagen gel where it appears brighter and stiffer in (D) at the top boundary of the gel and softer inside of the gel.

References:

1 Schneider, C. A., Rasband, W. S. & Eliceiri, K. W. NIH Image to ImageJ: 25 years of image analysis. *Nat. Meth.* **9**, 671-675 (2012).

2 Sokolov, I., Kalaparthi, V., Kreshchuk, M. & Dokukin, M. E. On averaging force curves over heterogeneous surfaces in atomic force microscopy. *Ultramicroscopy* **121**, 16-24, doi:10.1016/j.ultramic.2012.06.014 (2012).
